# Periocular rejuvenation using a unique non-ablative long-pulse 2940 nm Er:YAG laser

**DOI:** 10.1007/s10103-021-03362-6

**Published:** 2021-06-19

**Authors:** Ashraf Badawi, Tarek Sobeih, Vesel Jasmina

**Affiliations:** 1 Medical Laser Applications, National Institute of Laser Enhanced Sciences, Cairo University, Giza, Egypt; 2 Dermatology and Allergology Department, Faculty of Medicine, Szeged University, Szeged, Hungary; 3 Oakville, Canada; 4 Information Sciences Division, Nathan Kline Institute, Orangeburg, NY USA; 5 LA&HA - Laser and Health Academy, Ljubljana, Slovenia

**Keywords:** Non-ablative, Erbium YAG, Laser, Periocular, Wrinkles, Dark circles, Skin laxity, Rejuvenation, Periorbital rejuvenation

## Abstract

The periocular region is challenging for cosmetic laser surgeons. Surgery and laser resurfacing have traditionally been used to correct periorbital lines and wrinkles. Although effective, the associated downtime with these methods has made many people reluctant to decide for such treatments. More recently, the non-ablative long-pulse 2940 nm Er:YAG laser is being used to improve the structure and function and hence the appearance of skin in the periorbital region. The objective of this study is to evaluate the safety and efficacy of long-pulse 2940 nm Er:YAG laser for non-ablative treatment of periorbital static wrinkles and skin laxity. This is a prospective analysis of 30 patients treated for periorbital rejuvenation using three sessions of non-ablative long-pulse Er:YAG laser over a 3-month period. All patients were assessed according to Fitzpatrick’s classification of periorbital wrinkles to class I, II, or III and were treated with 2940 nm Er:YAG laser using a fluence of 3.75 J/cm^2^, a repetition rate of 1.7–2 Hz, and with the SMOOTH™ pulse mode (250 ms). The treatment sessions were performed on each patient, 4 weeks apart. Patient improvement was assessed before each laser session as well as at 12 months after the final treatment. Blind photographic evaluations were performed by three independent physicians using unlabeled before and after photos arranged in non-chronological order. Reviewers were asked to determine the before and after photos. Patients were asked to answer a questionnaire measuring satisfaction 4 weeks after each session, and to report any adverse reactions. There was statistically and clinically significant improvement in the Fitzpatrick classification of the periorbital wrinkles. Blinded evaluators correctly identified the before and after photos in all cases. All patients reported mild edema and erythema, which persisted for 1 to 2 days, and superficial peeling of the skin for 4 to 6 days after each laser treatment. No long-term adverse effects were reported. The non-ablative long-pulse 2940 nm Er:YAG laser seems to be a safe and effective treatment for periocular rejuvenation with minimal and tolerable adverse reaction. The improvement attained from the laser sessions was persisting after 1 year denoting the long-term efficacy of the procedure.

**Supplementary Information:**

The online version contains supplementary material available at 10.1007/s10103-021-03362-6.

## Introduction

Eye contact is the most powerful mode of establishing a communicative link between humans. The eyes play an essential role in non-verbal communication, which is why they are often the main focus of improving one’s aesthetic appearance. The eyelid skin is the thinnest in the body, and a loss of collagen means that wrinkles and fine lines appear around the eyes first[1].

The treatment of facial wrinkles using ablative lasers has been well documented[2]. Despite excellent results, they have been associated with considerable disadvantages such as pain, crusting, swelling, infections due to a prolonged recovery period, long-lasting erythema, and potential complications such as pigmentary changes and scarring[3–5]. For several years, researchers have been looking for non-invasive methods that will allow the deposition of thermal energy in the dermis in a way that triggers collagen remodeling and neocollagenesis while sparing the epidermis from injury[6].

The development of the variable-pulse Er:YAG laser for skin rejuvenation has demonstrated a potential application of this treatment approach [7]. In particular, the innovative Er:YAG SMOOTH™ mode is a non-ablative, non-invasive laser modality used for treatment of mucosa and skin. Previous studies have demonstrated that the SMOOTH™ mode modality has been used in the treatment of vaginal relaxation syndrome, stress urinary incontinence, and in onycomycosis[8–13]. The positive results of this laser on vaginal mucosal tissue have subsequently led to its intraoral use for the treatment of nasolabial folds (NLFs) and wrinkles. Oral and maxillofacial surgeons have also noticed improvement of perioral and facial rhytides on repeated usage of intraoral Er:YAG laser [14–18]. Additionally, a recent study presented a treatment option using non-ablative Er:YAG SMOOTH™ mode with a trans-conjunctival approach to tighten the periorbital skin below the eye, resulting in wrinkle and eye bag improvement [19].

The aim of this study was to evaluate the efficacy and safety of non-ablative long-pulse 2940 nm Er:YAG laser for the treatment of periorbital static wrinkles and skin laxity.

## Materials and methods

This prospective cohort study was approved by the local ethics committee at the National Institute of Laser Enhanced Sciences, Cairo University, Egypt (Registration number: Cu – NILES/01/21) and was conducted according to the guidelines of the Helsinki Declaration. All patients provided written informed consent. Adult patients with various degrees of wrinkles and lines in the periocular area were included in the study. In total, 30 patients (4 males and 26 females) between 33 and 73 years of age (mean ± SD = 46.8 ± 8.4) were enrolled in the study and completed three sessions of treatment. The exclusion criteria were any injectable treatment within the past year in the periorbital region, oculoplastic surgery within the past 2 years, or any topical treatment of the periorbital region other than the prescribed topical creams. The patients were instructed to avoid any of the above-mentioned procedures during the treatment period as well as the 1-year follow-up period after the final treatment session.

Er:YAG 2940 nm laser (SP Dynamis, Fotona, Ljubljana) was used with non-ablative SMOOTH™ mode pulses delivered via a patterned PS03 handpiece with 7-mm spot size, 3.75 J/cm^2^ fluence, and 1.7–2 Hz. Laser pulses were first applied on the upper eyelid followed by the lower eyelid. Three stacking pulses were performed per each spot followed by one painting pass covering the whole eye lid. The same technique was applied on both eyelids. Prior to each treatment, topical anesthesia was applied around the eyes for 30 min avoiding direct contact with the eyes. The topical anesthesia used was a locally compounded BLT (20% benzocaine, 6% lidocaine, 4% tetracaine with an emollient base). After 30 min of applying the topical anesthesia, the product was gently wiped off with a wet gauze to ensure that the skin is clean and dry to avoid any interference with the laser effect on the skin. During the procedure, the untreated eye was protected with a wet gauze held by the patient’s hand. In addition, the untreated lower eyelid was also covered with a wet gauze for protection. After finishing the upper lid of one eye, patients were asked to open their treated eye and look up to apply the wet gauze just below the lower eyelid lashes; then the patients were asked to close their eye to cover the treated upper eyelid with the wet gauze to start treating the lower eyelid. The untreated eye remains covered with the wet gauze all through this procedure. After finishing one side, we asked the patients to cover the treated eye with the wet gauze and repeat the procedure on the opposite side eye. The treated skin was stretched during laser application to achieve good optical penetration. After the treatment, a moisturizer cream containing panthenol was gently applied on the treated area. Patients were encouraged to use a moisturizer 3–4 times per day after each treatment in case of any superficial peeling. Each patient received three treatments, with a 4-week interval.

The patients were photographed before each session and 1 year after the final session after receipt of the required signed consents. The clinical outcome was evaluated by (A) the investigator’s (AB) evaluation, (B) the blinded evaluation, and (C) the patients’ self-evaluation.

A. The investigator’s evaluation

Wrinkles were evaluated based on the Wrinkle Assessment Scale that was developed by Lemperle[20] to assess wrinkle depth. The scale consists of six grading scores: 0= no wrinkles, 1 = just perceptible wrinkles, 2 = shallow wrinkles, 3 = moderately deep wrinkles, 4 = deep wrinkles, well-defined edges, 5 = very deep wrinkles, redundant fold. Wrinkling and degree of elastosis were assessed with the Fitzpatrick Wrinkle Classification System (FWCS)[21], which defines three classes of wrinkles: I. mild, II. moderate, and III. severe. Each of the three classes provides an additional three sub-scores for an overall scale extending from 1 to 9 (Table 1).

B. The blinded evaluation

**Table 1:** Fitzpatrick’s classification of facial wrinkling (perioral and periorbital)[20]

Class	Wrinkling	Score	Degree of elastosis
I	Fine wrinkles	1–3	Mild (fine textural changes with subtly accentuated skin)
II	Fine to moderate depth wrinkles, moderate number of lines	4–6	Moderate (distinct popular elastosis, individual papules with yellow translucency, dyschromia)
III	Fine to deep wrinkles, numerous lines, with or without, redundant skin	7–9	Severe (multipapular and confluent elastosis, thickened yellow and pallid cutis rhomboidalis)

Blinded evaluation of the before treatments and 1 year after last laser session photographs of the patients by three independent physicians. The photographs were given to them in a random order. The evaluators were asked to determine, the before and after image from a pair of photographs.

- C.Patients’ self-evaluation

Patients rated their satisfaction based on a 5-degree scale: − 1: worsening, 0: no improvement, 1: mild improvement, 2: moderate improvement, 3: excellent improvement.

The patients also reported on the following potential side effects: edema, erythema, and skin peeling (presence/absence and duration) and any possible additional side effects. Side effects were reviewed on the first visit following each treatment session.

### Statistical analysis

Statistical analyses were performed with GraphPad Prism 9.0.0 (GraphPad Software, Inc., San Diego, CA). Significance was assessed by repeated measures analysis of variance with Tukey’s post hoc analysis for continuous variables. All tests were two-tailed. P values less than 0.05 were considered significant.

## Results

All 30 patients completed three sessions, and 28 patients (93%) came to the 12-month follow-up visit. The mean value of the Lemperle score at baseline was 4, which was reduced to 3.4 at 4 weeks after the first session, and then to 2.6 at 4 weeks after the second session (adjusted *p* < 0.0001). Twelve months following the third session, the improvement was still maintained at a score of 2.5. All patients showed significant improvement (adjusted *p *< 0.0001) in the Lamperle Score between baseline and the 12-month post-treatment assessment (total 28 patients) (Table 2, Fig. 4).

**Table 2:** Lemperle score (for evaluation of wrinkle depth improvement)

	Baseline (*N* = 30)	After session 1 (*N* = 30)	After session 2 (*N* = 30)	12 months after session 3 (*N* = 28)
Lemperle score				
0	0	0	0	0
1	0	0	2 (6.7%)	2 (7.1%)
2	2 (6.7%)	4 (13.3%)	11 (36.7%)	14 (50.0%)
3	4 (13.3%)	15 (50.0%)	13 (43.3%)	9 (32.1%)
4	16 (53.3%)	7 (23.3%)	4 (13.3%)	3 (10.7%)
5	8 (26.7%)	4 (13.3%)	0	0
Mean Lemperle score (SD)	4.0 (± 0.8)	3.4 (± 0.9)*	2.6 (± 0.8)*	2.5 (± 0.8)*

^*^Defines statistical significance (*p* < 0.05) between Lemperle scores at follow-up and baseline

The mean value of the Fitzpatrick Score at baseline was 7, which was reduced to 5.1 at 4 weeks after the first session, then to 3.9 at 4 weeks after the second session (adjusted *p *< 0.0001). Twelve months following the third session, there was a non-significant improvement to 3.5 (Table 3). All patients showed significant improvement (adjusted *p* < 0.0001) in the Fitzpatrick Score between baseline and the 12-month post-treatment assessment (total 28 patients) (Table 3, Fig. 4).

**Table 3:** Fitzpatrick class and Fitzpatrick score

	Baseline (*N* = 30)	After session 1 (*N* = 30)	After session 2 (*N* = 30)	12 months after session 3 (*N* = 28)
Fitzpatrick class				
I	0	0	16 (53.3%)	16 (57.1%)
II	14 (46.7%)	23 (76.7%)	14 (64.7%)	12 (42.9%)
III	16 (53.3%)	7 (23.3%)	0	0 (0%)
Mean Fitzpatrick score (SD)	7.0 (± 1.4)	5.1 (± 1.2)	3.9 (± 1.2)	3.5 (± 1.1)

The changes in Fitzpatrick class were statistically significant (*P* < 0.0001) (Table 3, Fig. 4). A total of 26 patients showed an improvement in the Fitzpatrick class between baseline and the 12-month post-treatment assessment, while two patients showed no change (both subjects showed an improvement in Fitzpatrick score that was not enough to change their class).

In order to evaluate the long-term durability of the treatment, we evaluated the change in the Fitzpatrick class between the last treatment session and the 12-month post-treatment follow-up: two patients showed further improvement, 25 remained the same, and 1 showed a worsening in the Fitzpatrick class. The overall change was not statistically significant.

All the three blinded evaluators were able to successfully identify the before and after photos of all patients successfully.

The patients’ evaluation scores are summarized in Table 4. One patient observed a worsening of wrinkles at the 12-month follow-up, but with noted improvement by the clinician.

**Table 4:** Patient evaluation of treatment outcome

	After session 1 (*N* = 30)	After session 2 (*N* = 30)	12 months after session 3 (*N* = 28)
Satisfaction			
− 1: worsening	0	0	3.6%
0: no improvement	0	0	0
1: mild improvement	6.7%	3.3%	7.1%
2: moderate improvement	46.7%	46.7%	42.9%
3: excellent improvement	46.7%	50.0%	46.4%
Mean patient satisfaction score (SD)	2.4 (± 0.6)	2.5 (± 0.6)	2.3 (± 0.9)

**Fig. 1:**
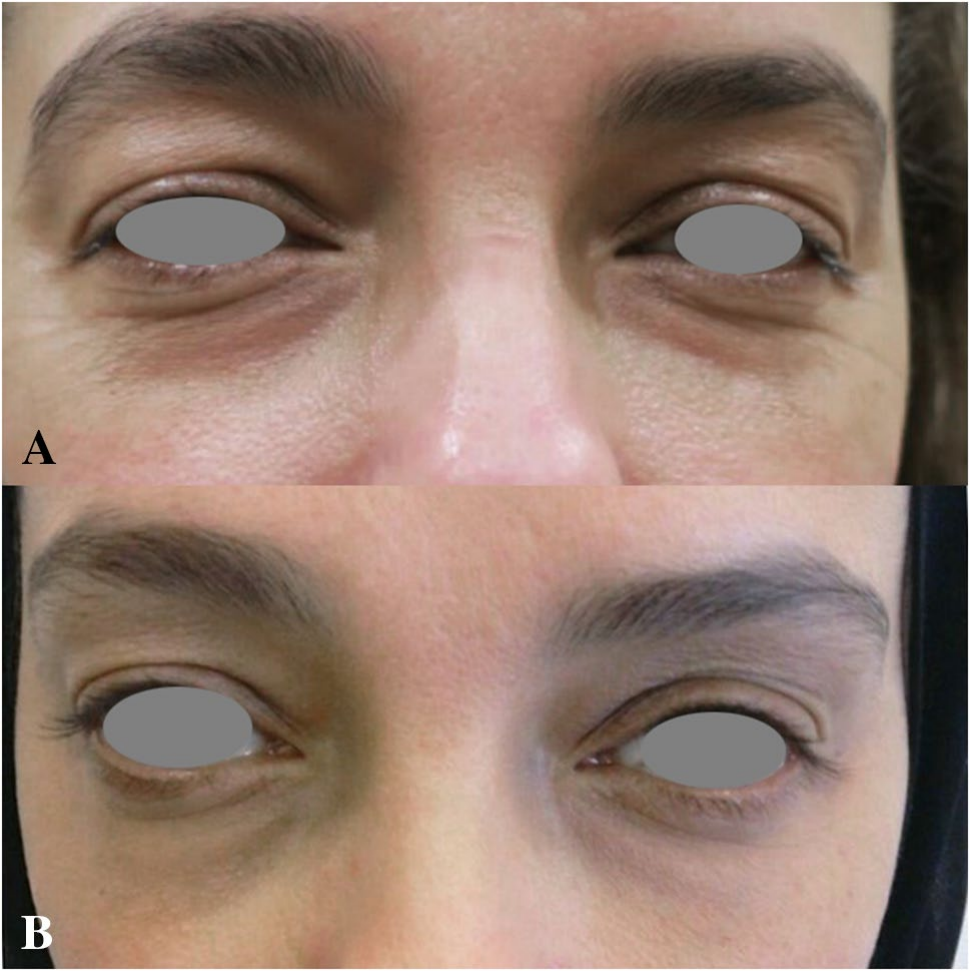
Improvement of periorbital wrinkles: (**a**) before treatment; (**b**) 12 months after the 3rd treatment

**Fig. 2:**
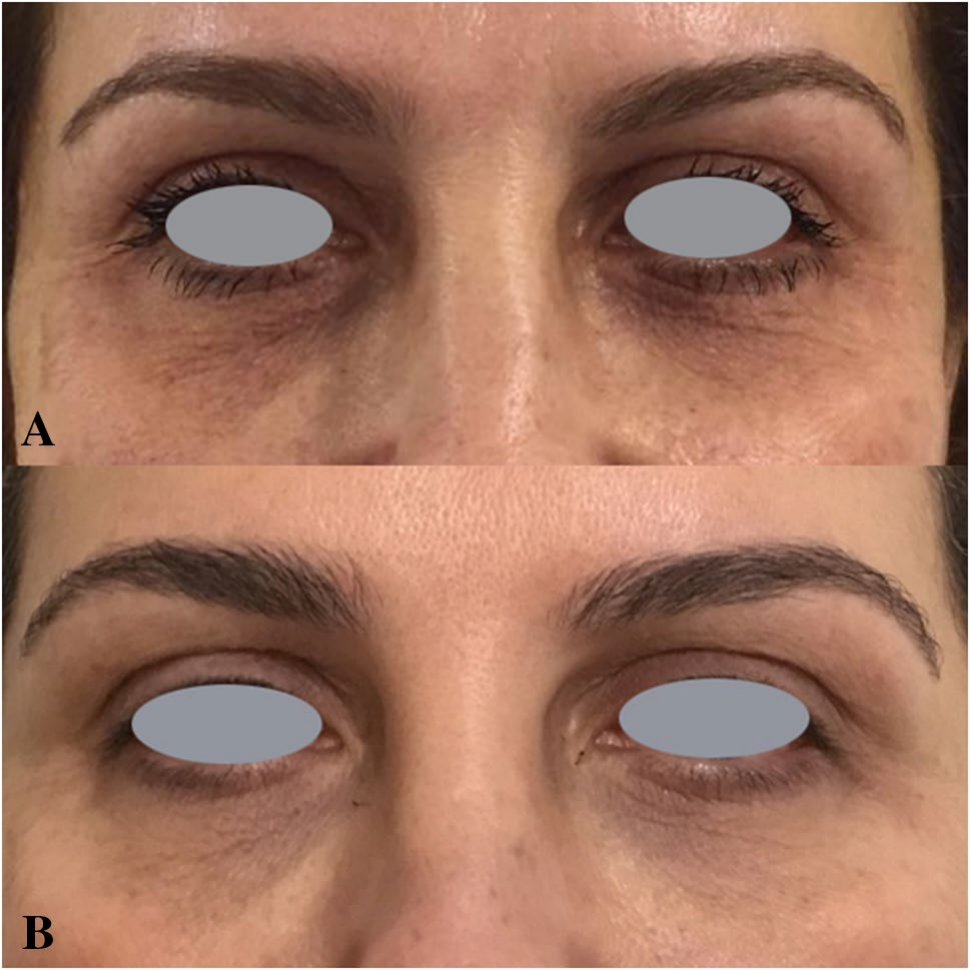
Improvement of periorbital wrinkles: (**a**) before treatment; (**b**) 1 year after last treatment

**Fig. 3:**
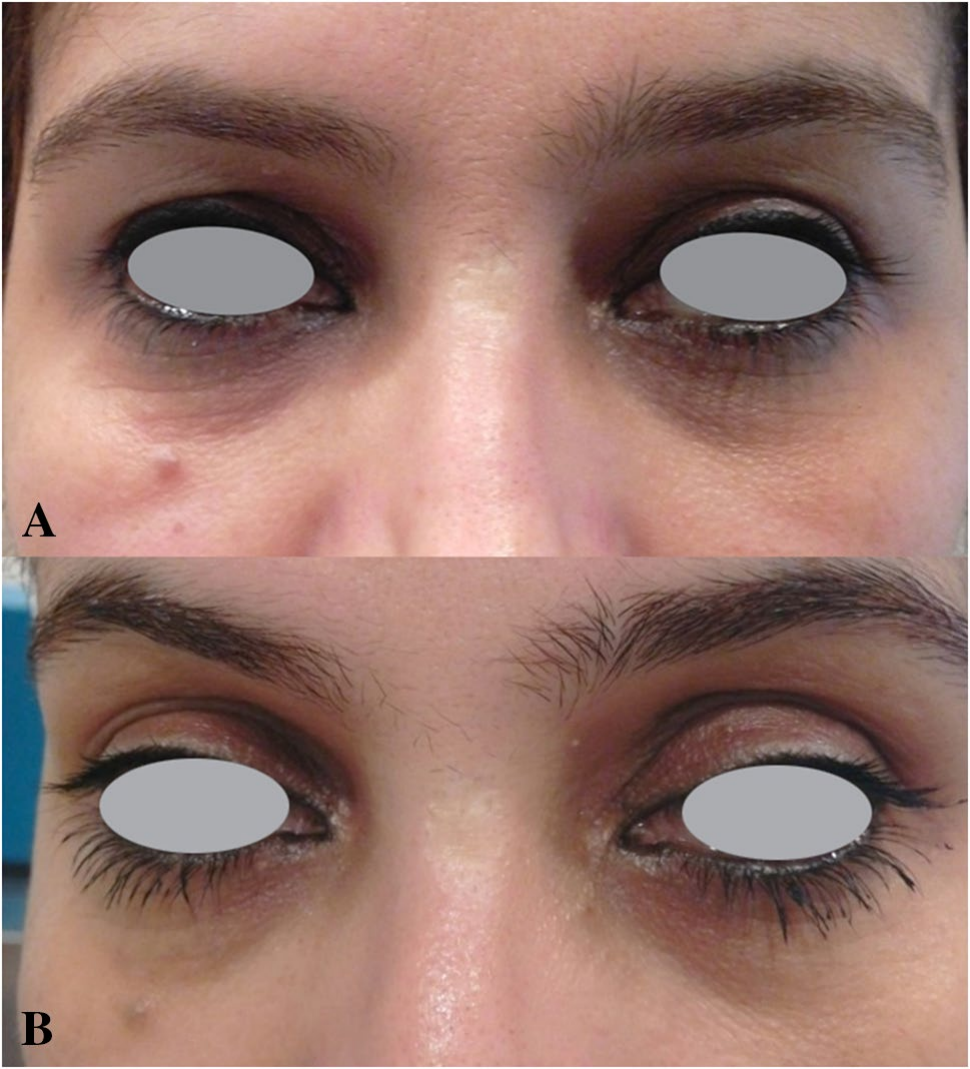
Improvement of periorbital wrinkles: (**a**) before treatment; (**b**) 1 year after last treatment

Photographic examples of clinical outcomes are demonstrated in Figs. 1, 2, and 3.

### Adverse effects

**Fig. 4:**
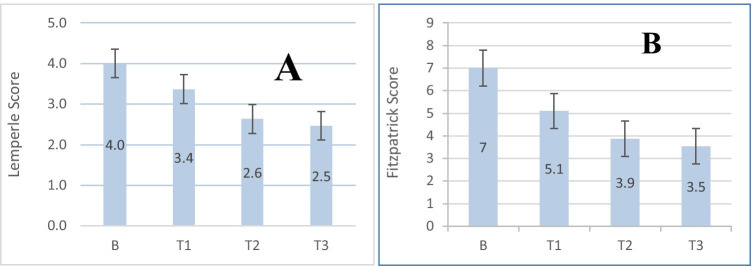
**A** Mean values of Lemperle Score and (**B**) Fitzpatrick Score at baseline, after 1st, 2nd, and 3rd sessions. B—baseline, T1—after 1st session, T2—after 2nd session, T3—after 3rd session

The number and duration of side effects are shown in Table 5. As reported by patients, erythema and or edema persisted for up to 12 h after the procedure. Erythema and edema were reported by 13.3–93.3% of patients after each session (Fig. 4). Skin peeling was observed after each session in most cases and persisted for up to 5 days. Skin peeling was reported by most patients after each session (93.3–100%). All the adverse effects were mild and resolved without permanent skin changing and scarring. No additional side effects were reported.

**Table 5:** Patient report on side effects presence and mean duration

Side effect	1st treatment N (%)	2nd treatment N (%)	3rd treatment N (%)	Range/mean duration
Erythema	28/30 (93.3)	27/30 (90)	27/30 (90)	(2–12 h)/5.5
Edema	19/30 (63.3)	15/30 (50.0)	11/30 (36.7)	(1–12 h)/3.8
Skin peeling	28/30 (93.3)	30/30 (100)	29/30 (96.7)	(2–6 days)/4.0

## Discussion

The present study showed an improvement in wrinkle depth and general wrinkling and degree of elastosis, which persisted for 12 months after 3 monthly treatment session. This study demonstrates that the use of non-ablative long-pulse Er:YAG laser for the treatment of periorbital wrinkles is a method with minimal downtime and a high satisfaction rate.

Many treatment modalities have been developed to improve the aesthetic appearance of periorbital areas showing signs of aging. The ablative lasers, 10,600-nm CO_2_ and 2940-nm Er:YAG, are considered the gold standard for periorbital rejuvenation. Laser resurfacing induces a controlled skin injury, with removal of the epidermis and variable portions of the dermis. Associated dermal heating results in collagen shrinkage and collagen remodeling [5, 22, 23]. Despite excellent results, they have been associated with many considerable disadvantages such as pain, crusting, swelling, infections as well as prolonged recovery period, long-lasting erythema, and potential complications such as pigmentary changes and scarring[5, 24]. Because of the long recovery time and side effects from ablative lasers, ablative lasers have become a less popular option and led to an increasing demand for the development of new modalities for skin rejuvenation as the non-ablative and fractional lasers which are associated with less downtime and minimal risks [22, 25].

Non-ablative lasers target tissues in the dermis by selective photothermolysis to stimulate collagen and dermal remodeling to reduce acne scar appearance[22]. Although they can be effective, these laser wavelengths have usually much higher optical tissue penetration compared to laser wavelengths that are used for ablative procedures — this is a disadvantage for treatments in the periocular area, as treatments with deeply penetrating wavelengths are not advised for safety reasons. On the contrary, the extremely short optical penetration depth of the 2940 nm Er:YAG laser makes it ideal to be used around the eyes. While the optical penetration of the long-pulse Er:YAG laser reaches only few microns when used with the appropriate fluence, tissue get non-ablatively heated to a depth of a few hundred microns, which is still much less than the thickness of the skin covering the eyelid[1, 26]. Recently, a non-ablative Er:YAG SMOOTH™ technology has been developed for minimally invasive treatments in aesthetics. It is based on a concept of controlled heating of the tissue with the objective to increase the temperature to 60–63 °C in short, microsecond pulses, which are arranged in optimally spaced pulse trains. This causes a dual regeneration effect on the tissue — short, microsecond heat pulses act as triggers of regenerative paracrine signaling pathways, enabling tissue turnover and regeneration, while the bulk heating of tissue causes immediate contraction of existing collagen and long-term stimulation of collagen formation [13, 27, 28]. The result is better quality and appearance of the skin, with subtle changes that improve for several weeks and months after the treatment.

This is the first prospective trial evaluating the safety and efficacy of Er:YAG laser used in non-ablative SMOOTH™ mode for the treatment of periocular wrinkles. This mode enables gentle heating of the skin and underlying tissue without any significant ablation of the superficial layers, and is especially suitable for sensitive periocular skin.

Our results showed that a series of three treatments delivered at 4-week interval was effective for improving periocular wrinkles. Improvement was noticed after each session and was maintained for up to 12 months after the final session. These results indicate that the effect is long-term, which may result from stimulation of the collagen remodeling process and neocollagenesis that continues after the treatment sessions has been completed. The effectiveness was confirmed with multiple scales. Evaluation based on the Fitzpatrick scale showed improvement in wrinkling; Fitzpatrick score demonstrated degree of elastosis and Lemperle score improvement in wrinkle depth. The results were very satisfactory in most patients. One patient observed worsening of the wrinkles, which was not in line with the clinician’s observation. This can be attributed to a gradual and slow improvement over a period of time, during which the patient might forget the pre-treatment look. Thus, it is very important to document the pre-treatment condition with photographs. The correct assessment of 100% of the blinded before-and-after photographs by the three independent dermatologists further confirmed the clear effectiveness of this treatment method. Our study confirms previous published clinical data studying this unique laser modality on other anatomical locations, as summarized below.

Non-ablative Er:YAG laser with SMOOTH™ mode has been previously shown to be effective in the treatment of mucosal tissues of the vaginal canal for treating stress urinary incontinence, vaginal laxity, and vaginal atrophy. It was also recently found effective in treating nasolabial folds (NLFs) with an intraoral approach[8–10, 13]. The efficacy of the intraoral fractional Er:YAG SMOOTH™ mode in rejuvenation of NLFs has been investigated in a few non-comparative studies with promising results[16–18]. A prospective randomized split-face comparative pilot study investigated the safety and efficacy of the intraoral approach of Er:YAG laser SMOOTH™ mode and an extraoral approach in the rejuvenation of NLFs (4 J/cm^2^, five sessions with 4-week interval). A comparison between the intra- and extra-oral sides using optical coherence tomography (OCT) evaluation of both epidermal and dermal thickness showed a significantly thicker dermis in the intraorally treated sides. The thickness increased by 29% compared to the baseline on the intraoral sides and 2% on the extra-oral side. However, patients were significantly more satisfied with the extra-oral approach at 2 weeks and 4 months after the final session[14]. A study of Serdar et al. [29] compared Er:YAG non-ablative SMOOTH™ mode to fractional radiofrequency (fRF) for treatment of the neck and submental regions. Both treatments were shown to be similarly effective: 89–93% of patients treated with Er:YAG SMOOTH™ mode declared themselves to be satisfied or very satisfied compared to 86–92% treated with fRF. However, in the same study, the periorbital area was treated with fRF or ablative fractional Er:YAG (AFR Er:YAG), and patient satisfaction was much lower for fRF: 61% of patients were satisfied or very satisfied after fRF, compared to 82% after AFR Er:YAG.

Kim et al.[19] presented a novel method where non-ablative Er:YAG SMOOTH™ mode is used over the lower eyelid conjunctiva. The treatment consisted of 12 non-overlapping passes with increasing fluence from 3 to 4.5 J/cm_2_, 1.8 Hz, and the number of stacks from two to five. Three treatment sessions with 2-week intervals resulted in a great reduction of the volume of eye bags and the severity of wrinkles below the eye. One month after the treatment, 87% of patients were satisfied (moderate to excellent)[19]. Studies of Majaron et al.[30] and Drnovšek-Olup et al.[31] revealed that deep collagen remodeling and new collagen synthesis occur as a results of the SMOOTH™ mode treatment, with less epidermal damage compared to standard Er:YAG laser skin resurfacing. New collagen synthesis after Er:YAG laser skin resurfacing in eyelid skin was further confirmed by in situ hybridization (ISH)[32].

Collagen types I, III, and VII, as well as newly synthesized collagen, together with tropoelastin showed a statistically significant increase in response to SMOOTH™ mode mini-peels (2–3 passes at 2–3 J/cm^2^), while the mean level of total elastin was significantly decreased in response to treatment[33]. This was followed by regression of improvement at 3 months post-treatment, but this was still better than baseline[33].

Manuskiatti et al.[7] evaluated the effects of low-fluence Er:YAG laser pulse widths on the treatment outcome of periorbital wrinkles in Asian subjects. In the short pulse (SP) mode, the patients were treated with two passes with 50% overlap of Er:YAG laser using a fluence of 0.5 J/cm^2^, giving a total of up to 1 J/cm^2^ with an ablation depth of approximately 4.5–6.0 m$\mu$/J/cm^2^. In the super-long-pulse (SL = SMOOTH) mode, the sub-ablative laser energy was delivered in an overall pulse duration of 250 ms, consisting of six equally spaced pulses with a repetition rate fixed to 1 Hz. The study demonstrated that low-fluence, variable square pulse (VSP) Er:YAG effectively improved periorbital wrinkles in skin phototypes IV–V with minimal downtime and low risk of transient adverse effects. The study showed that for the subjects with mild to moderate periorbital wrinkles, there was no significant difference in the treatment outcome when comparing the SP and SL groups. This observation was confirmed by the patients’ self-evaluation, reporting comparable improvement grading in the SP and SL groups at the 3-month follow-up visit.

Philips[34] reported a very successful periocular rejuvenation in one case where the same SMOOTH™ mode as in our study was used. Three sessions every 6 weeks were applied with 3.5 J/cm^2^ with 3–4 pulse trains per each spot. Great wrinkle improvement was observed at follow-up 8 weeks after the final treatment.

Improved wrinkle appearance that was evident from the results of our study could be attributed to improved skin elasticity, which has previously been proved to be achieved using the same SMOOTH™ laser modality[35].

Our study has also clearly demonstrated the safety of this therapy. None of the patients experienced any long-lasting side effects. Minimal side effects such as erythema and edema spontaneously resolved in a matter of hours. Skin peeling completed in few days. Short downtime and very low incidence of side effects is a major advantage over other periorbital rejuvenation methods.

Er:YAG SMOOTH™ mode is based on the principle of restoring the structure and function of the skin; its improvement is achieved by structural improvement over several months after initiating the treatment. This is considered as a major advantage over the soft tissue fillers which are associated with minimal improvement of the skin structure as well as high incidence of complications.

Limitations of this study include lack of follow-up at four weeks after 3rd session to compare the improvement after 3rd session versus after the 2nd session. If that was included in our study, we would have been able to demonstrate if there was a significant difference between the wrinkle improvement after the second session compared to that of the third session. Another limitation was the absence of a control group to further demonstrate the safety and effectiveness of this method. The lack of objective evaluation method for the periocular skin was an additional limitation of our study due to the lack of budget to have an objective evaluation tool.

Patient education, expectation management, and regular follow-ups with at least photographic assessment are important to achieve the highest level of patient satisfaction.

## Conclusion

The non-ablative SMOOTH™ mode with 2940 nm Er:YAG laser seems to be a safe and effective procedure for periocular rejuvenation. The method showed excellent immediate and long-term results, with minimal downtime and neglectable adverse effects. Non-ablative long-pulse Er:YAG treatment might also serve as a preventive measure to delay signs of early aging.

## Supplementary Information

Below is the link to the electronic supplementary material.

Supplementary file1 (XLSX 50 KB)
